# Resting state EEG in youth with ASD: age, sex, and relation to phenotype

**DOI:** 10.1186/s11689-021-09390-1

**Published:** 2021-09-13

**Authors:** Emily Neuhaus, Sarah J. Lowry, Megha Santhosh, Anna Kresse, Laura A. Edwards, Jack Keller, Erin J. Libsack, Veronica Y. Kang, Adam Naples, Allison Jack, Shafali Jeste, James C. McPartland, Elizabeth Aylward, Raphael Bernier, Susan Bookheimer, Mirella Dapretto, John D. Van Horn, Kevin Pelphrey, Sara Jane Webb, and the ACE GENDAAR Network

**Affiliations:** 1 Center on Child Health, Behavior and Development, Seattle Children’s Research Institute, 1920 Terry Ave, CURE-03, Seattle, WA 98101 USA; 2 Department of Psychiatry and Behavioral Sciences, University of Washington, Seattle, USA; 3 Mailman School of Public Health, Columbia University, New York, USA; 4 School of Medicine, Emory University, Atlanta, GA USA; 5 Marcus Autism Center, Children’s Healthcare of Atlanta, Atlanta, GA USA; 6 Division of Developmental Medicine, Department of Medicine, Boston Children’s Hospital, Boston, USA; 7 Department of Psychology, Stony Brook University, Stony Brook, USA; 8 Department of Special Education, University of Illinois at Chicago, Chicago, USA; 9 Yale Child Study Center, Yale University, New Haven, USA; 10 Department of Psychology, George Mason University, Fairfax, USA; 11 Department of Psychiatry & Biobehavioral Sciences, University of California Los Angeles School of Medicine, Los Angeles, USA; 12 Intellectual and Developmental Disabilities Research Center, University of California Los Angeles, Los Angeles, USA; 13 Center for Integrative Brain Research, Seattle Children’s Research Institute, Seattle, USA; 14 Department of Psychology, University of Virginia, Charlottesville, USA; 15 School of Data Science, University of Virginia, Charlottesville, USA; 16 Department of Neurology, Brain Institute and School of Education and Human Development, University of Virginia, Charlottesville, USA; 17 Intellectual and Developmental Disabilities Research Center, University of Washington, Seattle, USA

**Keywords:** Autism, Biomarker, EEG, Electroencephalography, Resting, Alpha, Power, Sex differences

## Abstract

**Background:**

Identification of ASD biomarkers is a key priority for understanding etiology, facilitating early diagnosis, monitoring developmental trajectories, and targeting treatment efforts. Efforts have included exploration of resting state encephalography (EEG), which has a variety of relevant neurodevelopmental correlates and can be collected with minimal burden. However, EEG biomarkers may not be equally valid across the autism spectrum, as ASD is strikingly heterogeneous and individual differences may moderate EEG-behavior associations. Biological sex is a particularly important potential moderator, as females with ASD appear to differ from males with ASD in important ways that may influence biomarker accuracy.

**Methods:**

We examined effects of biological sex, age, and ASD diagnosis on resting state EEG among a large, sex-balanced sample of youth with (*N* = 142, 43% female) and without (*N* = 138, 49% female) ASD collected across four research sites. Absolute power was extracted across five frequency bands and nine brain regions, and effects of sex, age, and diagnosis were analyzed using mixed-effects linear regression models. Exploratory partial correlations were computed to examine EEG-behavior associations in ASD, with emphasis on possible sex differences in associations.

**Results:**

Decreased EEG power across multiple frequencies was associated with female sex and older age. Youth with ASD displayed decreased alpha power relative to peers without ASD, suggesting increased neural activation during rest. Associations between EEG and behavior varied by sex. Whereas power across various frequencies correlated with social skills, nonverbal IQ, and repetitive behavior for males with ASD, no such associations were observed for females with ASD.

**Conclusions:**

Research using EEG as a possible ASD biomarker must consider individual differences among participants, as these features influence baseline EEG measures and moderate associations between EEG and important behavioral outcomes. Failure to consider factors such as biological sex in such research risks defining biomarkers that misrepresent females with ASD, hindering understanding of the neurobiology, development, and intervention response of this important population.

## Introduction

Autism spectrum disorder (ASD) is a neurodevelopmental disorder defined by social communication impairments and repetitive behaviors [1], but also by extreme heterogeneity in symptom severity, cognitive functioning, comorbid conditions, and medical involvement. Increasingly, the field of ASD research has sought to identify biomarkers with specificity for ASD, with multiple goals of characterizing brain systems associated with its etiology, contributing to diagnostic clarification, delineating subgroups within the larger ASD population, and monitoring change due to intervention and developmental processes [2–5]. Within these efforts, electroencephalography (EEG) carries tremendous promise, as it is well tolerated by children with ASD [6], appropriate across a range of verbal and attentional skills, and suitable for multisite assessment [5].

### EEG in ASD

EEG provides a method of understanding both resting neural activation and task-engaged states in autism (e.g., [7, 8]; for review, see [8]) and is a multiplexed signal that reflects activity of pyramidal neurons in the neocortex [9]. This neurophysiological activity can be decomposed into frequency bands (ranging from 0.3 to 100 Hz) that reflect both state and trait functioning in cognitive and clinical domains (for a review of EEG generation and functional role, see [10]).

In brief, low frequency slow-wave oscillations in the delta band (1–4 Hz range) have been implicated in resting state cortical activity during early infancy. Later in development, delta slow waves are related to event-related detection of attention, salience, and motivation (e.g., [11]), with differences in delta power observed in neurodevelopmental concerns such as ADHD [12], dyslexia [13], and preterm birth [14]. Theta waves (4–8 Hz range) are thought to reflect cognitive activities such as focused attention, effort, stimulus processing, memory, and recall, and are largest at frontal-central regions [10]. Theta differences have been associated with clinical concerns including Fragile X [15], ADHD [16], and psychosis risk [17]. Alpha waves (8–13 Hz range) are an inverse measure of brain activity, with greater alpha amplitude found during resting compared to wakefulness. Decreases in alpha activity are associated with cognitive states such as inhibition, attention, and sensory control [18, 19], as well as social understanding and imitation [20]. Beta waves (13–30 Hz range) are associated with task engagement, motor control, and general alertness [8, 21]. Lastly, gamma waves (30–80 Hz range) increase during sensory responses, working memory tasks, and feature binding [10, 22].

Given this wealth of developmental and cognitive correlates, studies seeking ASD biomarkers frequently investigate resting state EEG. However, associations between ASD and resting state EEG have not been straightforward. In a comprehensive review, Wang et al. [8] proposed that individuals with ASD demonstrate an overall shift in the EEG spectrum, resulting in an altered shape of the power profile. Relative to comparison groups, they suggest that those with ASD display increased power at lower frequencies (delta, theta) and higher frequencies (beta, gamma), but decreased power in the middle frequency range (alpha). Although intriguing, this pattern has not been consistently observed in the literature. For example, in one of the largest published samples to date, Chan et al. [23, 24] found that children with ASD had increased delta power relative to controls, but did not differ with regard to theta, alpha, or beta frequencies. Further, although Wang et al. [8] suggest topographically widespread differences in power in ASD, other studies indicate hemispheric differences in the form of increased power in left frontal, parietal and temporal regions [25–28]. A recent comprehensive review further underscores the variability observed within EEG findings among children with ASD [29].

### Relation to child characteristics

Given the heterogeneity of ASD, one source of variation in EEG findings may be individual differences among participants. Primary among these differences is participant age. Typical development is characterized by an age-related shift in EEG power by frequency over the course of childhood, such that slow-wave frequencies (delta and theta) decrease in amplitude with age, whereas alpha, beta, and gamma increase (e.g., [30–32]). Among youth with ASD, studies using magnetoencephalography (MEG) to assess oscillatory activity across comparable frequency bands suggest similar patterns of age-related change during childhood and early adolescence [33, 34].

Aspects of clinical phenotype may contribute to variation in EEG power as well, but links between ASD severity and resting state activity are inconsistent overall [29] and knowledge is largely limited to males with ASD. Among samples containing only or primarily males, higher levels of resting gamma activity have been associated with decreased ASD symptom severity [35], but also to greater developmental delays among two independent samples of boys with ASD [36]. Among adults with ASD, lower levels of posterior alpha activity related to stronger preferential attention to detail [37], often considered an ASD trait [38]. An additional study examining the overlap between ASD and ADHD found that higher levels of ASD symptoms corresponded to decreased delta, theta, and alpha power among a combined ASD and ADHD sample [12]. Whether findings extend to women and girls with ASD is largely unknown, but emerging longitudinal evidence suggests that sex may indeed moderate associations between EEG and ASD features. Specifically, Brito et al. [39] found that greater EEG power for higher frequencies during infancy predicted stronger social-emotional competence in toddlerhood for males, but no such relationship was found for females.

Despite the potential value of resting EEG as an ASD biomarker, its utility is hampered by several factors. First, study sample sizes have been quite small historically (e.g., including 30 or fewer participants), particularly when considering the heterogeneity they likely contain. Second, participant groups typically have very high male-to-female ratios among their participants with ASD (often consistent with diagnostic ratios), leading to underrepresentation of girls and women with ASD. While these concerns are not unique to EEG studies, they are particularly problematic with regard to biomarker research, as they raise questions as to whether ASD biomarkers carry equal validity for all individuals with ASD. To the extent that biomarkers inform conceptual and etiological models, diagnostic processes, and methods of prevention and intervention, identification of such discrepancies is essential.

### Current study aims

To better understand neurodevelopmental and sex-based differences in youth with ASD, we conducted a four-site study to enroll a large, sex-balanced sample of children aged 8 to 18 years with ASD or typical development. Within this sample, we investigated EEG power during an eyes open resting EEG experiment, abstracting power for the major frequency bands, and investigated the effects of diagnostic group, age, and sex. Following Wang et al. [8], we hypothesized that we would find reduced power in the alpha frequency, and increased power in delta, theta, beta, and gamma frequencies. We also expected to find main effects of age, with a shift toward higher frequencies across our age range.

Given the historic underrepresentation of female participants in ASD research, we also sought to understand whether associations between resting state EEG power and individual differences in phenotypic characteristics are consistent across males and females with ASD. To this end, we conducted exploratory analyses to investigate the relation between EEG power, and verbal and nonverbal cognitive ability, social skills, and restricted/repetitive behavior. These variables were selected in light of their potential value in biomarker studies, for which knowledge of sex-based differences is of particular conceptual and clinical importance.

## Methods

### Participants

Participants between the ages of 8 and 17 years (47% female) were recruited as part of the Autism Center for Excellence (ACE) project Multimodal Developmental Neurogenetics of Females with ASD (MH100028), focusing on sex differences in children with ASD. Of the enrolled children, 339 participated in the EEG protocol, and 280 provided artifact-free eyes open resting EEG data. Data loss was due to acquisition errors (*n* = 10) or data quality (e.g., artifacts, blinks; *n* = 49).

#### Recruitment

Data collection sites included Boston Children’s Hospital (BCH), Seattle Children’s Research Institute (SCRI), the University of California Los Angeles (UCLA), and Yale University, with the data coordinating center located at the University of Southern California (USC). Participants were recruited using flyers and recruitment lists using strategies that reflected best practices at each site. The study was approved by the research ethics committee or institutional review board at the local site and by the Yale IRB. Parents of participants provided consent and participants provided assent. All data were collected using a numerical identifier and personal health information was maintained by the data collection site only. De-identified data was provided to the National Database of Autism Research (NDAR study #2021).

#### Enrollment

For inclusion in the study, all participants were required to be between 8 years, 0 months and 17 years, 11 months. Participants were excluded if they were a twin, or if they had a history of known single-gene conditions related to ASD (e.g., Fragile X; assessed through parent-reported medical history), medical conditions likely to be etiological (e.g., focal epilepsy), history of active seizures within the past year, clinically significant visual or auditory impairment after correction, sensory-motor difficulties or active tic disorder that would preclude valid use of the diagnostic instruments or neuroimaging, or any neurological disorder involving pathology above the brainstem. Participants were also excluded if they had a history of significant prenatal/perinatal/birth injury or neonatal brain damage, gestation less than 36 weeks and birthweight less than 2000 grams, NICU hospital stay over 3 days, and any environmental circumstances that might account for behaviors related to autism (e.g., severe nutritional or psychosocial deprivation). We did not include participants currently using any benzodiazepine, barbiturate, or anti-epileptic medication, or participants with medication changes within the 6 weeks prior to enrollment.

#### ASD

Children in the ASD group (ASD) had a prior clinical and/or research diagnosis of ASD that was confirmed using the Autism Diagnostic Interview (ADI-R) [40], Autism Diagnostic Observation Schedule (ADOS-2) [41] and DSM-IV-R [42] as administered by a research reliable clinician. Participants with ASD met “autism spectrum” criteria on the ADOS-2 and “autism” criteria on the ADI-R within 2 points. An additional 7 participants were missing the ADI-R, and thus were qualified based on meeting criteria on the ADOS-2 and based on parent report using the Social Responsiveness Scale, 2nd edition (SRS-2) [43] and Social Communication Questionnaire (SCQ) [44], with scores in the ASD clinical range on both measures.

#### NT

Neurotypically (NT) developing children had no elevated report of autism symptoms via parent report on the SRS-2 (*T*-score < 60) or the SCQ (raw score < 11), as well as no clinician impression of ASD, and no first- or second-degree relatives with ASD. Youth in the NT group also had no diagnosis or behaviors suggestive of schizophrenia, learning/intellectual disability, or other developmental or psychiatric disorder via parent report.

#### Descriptive characterization

Table 1 provides descriptive data for participants. Cognition was measured using the Differential Ability Scales 2nd edition (DAS-II) School Aged Cognitive Battery [45] including the Verbal, Nonverbal, and Spatial reasoning standard scores (SS) and participants had to have at least one subtest standard score > 70. Additional assessment was done to quantify ASD phenotype via the SRS-2 [43] and adaptive skills via the Vineland Adaptive Behavior Scales, 2nd edition (Vineland-II) [46] for parent-reported socialization, communication, and daily living skills. As shown in Table 1, the ASD group was younger, had higher autism traits (SRS-2), and had lower cognitive, language, and adaptive skills (DAS-II, Vineland-II).

**Table 1:** Participant descriptive information. Mean (SD)

	ASD		NT		Diagnostic group effect
Total *N* = 280	Female, *N* = 61	Male, *N* = 81	Female, *N* = 68	Male, *N* = 70	*χ*^2^ (1) = 1.12, *p* = .289
Age (months)	149.51 (34.77)	146.51 (34.21)	157.99 (36.88)	161.37 (33.09)	*F*(1, 278) = 8.27, *p* = .004
ADOS-2					
Total CSS	6.51 (1.80)	7.16 (1.84)	--	--	--
SA CSS	6.61 (1.79)	7.28 (1.89)	--	--	--
RRB CSS	6.84 (2.59)	6.54 (2.59)	--	--	--
SRS-2					
Total (raw score)	95.71 (27.33)	90.38 (28.02)	17.31 (12.31)	15.74 (12.04)	*F*(1, 252) = 836.65, *p* < .001
Total (T-score)	78.22 (11.55)	72.60 (11.18)	45.09 (5.21)	42.86 (4.84)	*F*(1, 252) = 790.13, *p* < .001
DAS-II					
Verbal SS	102.69 (20.54)	99.53 (20.66)	112.72 (15.44)	113.12 (16.78)	*F*(1, 273) = 28.91, *p* < .001
Nonverbal reasoning SS	100.85 (17.64)	101.28 (17.86)	110.25 (15.92)	109.58 (14.80)	*F*(1, 273) = 19.36, *p* < .001
Spatial SS	98.20 (18.10)	99.91 (17.33)	109.87 (12.98)	111.41 (15.33)	*F*(1, 273) = 35.15, *p* < .001
Vineland-II					
Communication SS	78.35 (13.95)	75.88 (10.87)	101.45 (13.58)	96.87 (12.56)	*F*(1, 256) = 192.30, *p* < .001
Socialization SS	74.16 (13.48)	73.72 (10.92)	102.37 (13.27)	100.74 (11.46)	*F*(1, 256) = 328.83, *p* < .001
Daily living skills SS	78.65 (14.82)	75.94 (13.09)	100.29 (12.67)	95.58 (13.13)	*F*(1, 256) = 152.32, *p* < .001

*ADOS-2*, Autism Diagnostic Observation Scale, 2nd edition [41]; *SA*, social affect; *RRB*, restricted repetitive behavior; *CSS*, Calibrated Severity Score; *SRS-2*, Social Responsiveness Scale, 2nd edition [43]; *DAS-II*, Differential Ability Scales, 2nd edition [45]; *SS*, Standard Score; *Vineland-II*, Vineland Adaptive Behavior Scales, 2nd edition [46].

### EEG protocol

To maximize consistency across sites, all four data collection sites utilized the EGI 128 channel Net Amps 300 system with HydroCel nets (EGI Inc., Eugene OR). Timing tests were run monthly to ensure standard stimulus presentation time and identify any timing corrections that needed to be applied during post-processing. Nets were available without outriders (eye electrodes #125, 126, 127, and 128) for participants with facial sensory sensitivities. Nets were verified to be in good working order monthly and tracking of net repair was kept by the EEG data analytic core.

Data were collected using Net Station 4.4.2, 4.5.1, or 4.5.2 using a standard Net Station acquisition template^1^. Stimulus control utilized EPrime 2.0. Monitors differed by site, but all experiments were calibrated so that the visual angle of the stimuli was equivalent across sites. A Logitech speaker system X320 was used for auditory presentation; auditory levels were confirmed using a sound meter. The participant’s behavior was video recorded during EEG collection. Data was collected at 500 Hz, referenced to Cz (vertex), and impedances were < 50 KOhms.

#### Acquisition

Participants were allowed to familiarize themselves with the EEG space and to explore the equipment prior to participation in the EEG session. Social scripts were utilized as needed, and a behavioral assistant provided additional support and prompting when necessary to maintain experimental compliance. All experiment specific instructions were included in the EPrime experiment script and were presented both visually and aloud. Behavior was recorded on a study specific log form that characterized participant behavior by experiment block. On site staff coded participant behavior during the task; data for which the participant was talking, moving, or non-attentive was marked and discarded in post-processing.

The total EEG session was approximately 60 minutes in duration, with experiments conducted in a fixed order. Resting state EEG was assessed via an eyes open (EO) resting experiment, with the following introduction: “You are going to watch short movies and then rest your eyes.” The experiment consisted of three runs of 6 × 16 second blocks of videos (dynamic screen saver type images that had limited or slow movement) or eyes closed. The videos were displayed at a height of 7–8.5 cm × 9.4–10.6 cm and resulting in a visual angle of 5.5–6.7 to 7.5–8.5°. The videos were small in order to match the size of other stimuli in the battery and to reduce eye movements. Videos were standardized across all four study sites.

#### Signal processing

Resting EEG data were bandpass filtered at .1 and 100 Hz with a 60-Hz notch filter. Data were segmented in 2048 ms segments and data with poor attention and behavioral non-compliance were removed. Bad channels were identified as any electrode with a greater than 100 microvolt difference between its lowest and highest point. Segments with more than 24 bad electrodes (including eye electrodes) were removed. Manualized hand editing was then performed to remove segments with remaining significant eye movements and eye artifacts. Trials were rejected for eye artifacts if (1) the first two rim rows of electrodes (8, 9, 14, 15, 21, 22, 25) were all rejected by the Net Station 4.5.6 bad channel algorithm, or (2) the first two rows of electrodes were partially rejected by the bad channel algorithm and the third row of electrodes (3, 10, 16, 18, 23) still showed the morphology of a blink or eye artifact. On the remaining good trials, bad electrodes were replaced using the Replace Bad Channel Function. Data were re-referenced to an average reference. Participants were required to have 10 segments (20 s) with valid, artifact-free data to be included in the study. The amount of data retained differed by group (*F*(1, 276) = 22.67, *p* < .001). The NT group had more remaining artifact-free trials (*n* = 81.04, *SD* = 24.08) than the ASD group (*n* = 66.63, *SD* = 26.28).

#### Power derivation

**Fig. 1:**
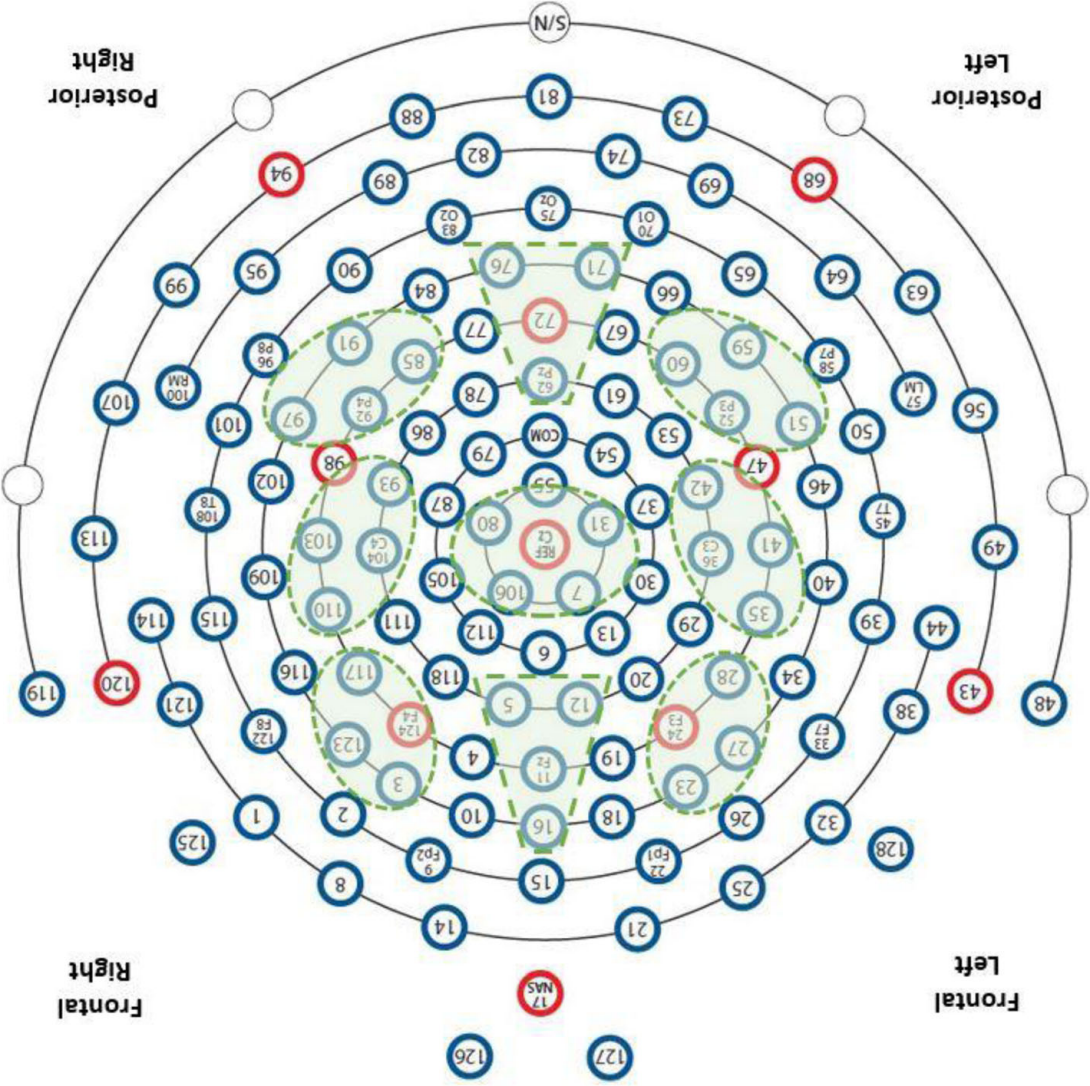
EEG montage with channels indicated. Channel numbers for regions are (1) frontal left (23, F3-24, 27, 28), (2) frontal midline (5, FZ-11, 12, 16), (3) frontal right (3, 117, 123, F4-124), (4) central left (35, C3-36, 41, 42), (5) central midline (7, 31, 80, 106), (6) central right (93, 103, C4-104, 110), (7) posterior left (51, P3-52, 59, 60), (8) posterior midline (Pz-62, 71, 72, 76), and (9) posterior right (85, 91, P4-92, 97)

We utilized 9 regions of interest (ROI); regions were selected so that left, midline, and right portions of frontal, central, and posterior scalp were represented during analysis. See Fig. 1 for a visual representation of regions and channels. The approximate 10–20 system electrode equivalents and the channel numbers for the regions are: (1) frontal left (23, F3-24, 27, 28), (2) frontal midline (5, FZ-11, 12, 16), (3) frontal right (3, 117, 123, F4-124), (4) central left (35, C3-36, 41, 42), (5) central midline (7, 31, 80, 106), (6) central right (93, 103, C4-104, 110), (7) posterior left (51, P3-52, 59, 60), (8) posterior midline (Pz-62, 71, 72, 76), and (9) posterior right (85, 91, P4-92, 97). The time-domain EEG signal was transformed to the frequency-domain using Welch’s power spectral density estimate in a custom Matlab script (PWELCH function, see [47, 48]). FFTs for each 1024-sample segment were calculated on 512-point Hamming windows with 50% overlap resulting in a 0.5 Hz frequency resolution. Absolute power was calculated for the delta (1–3 Hz), theta (4–7 Hz), alpha (8–12 Hz), beta (13–29 Hz), and gamma (30–50 Hz) frequency bands. Absolute power (μV^2^) was calculated by summing power estimates at every increment within each frequency band and then averaging across all electrodes within each ROI. To normalize the distribution, values were then natural logarithm-transformed.

### Statistical methods

Mixed-effects linear regression models using Stata v.14.2 (StataCorp, TX) were used to assess differences in power associated with age (continuous, in years), autism diagnosis, and sex. Nested random effects were included for site and family, to account for clustering by each (as there could be multiple participants within a family). A separate model was run for each frequency-region combination. Covariates included age, autism diagnosis group (ASD vs NT), and sex. We also sequentially tested for interactions between these covariates, first testing for 3-way interactions (age × sex × group) in each model; these were ruled out based on non-significant interaction terms. We then tested for and ruled out co-occurring two-way interactions (for example, sex × age and sex × group in the same model). Finally, individual two-way interactions were tested, and three were found to be significant and were thus retained in the corresponding models.

Beta coefficients corresponding to the predictors of interest (age, group, sex) were exponentiated to yield adjusted percent differences in power associated with the predictor of interest. Since outcome data were log-transformed, the original beta coefficient represents the difference in log power associated with a 1-unit change in predictor; to assess differences in terms of power rather than log power, exponentiating the beta coefficient yields the relative risk, which has been converted to a “percent difference” in power associated with a 1-unit change in predictor. For models with a significant interaction term, sex-specific percent differences were estimated. All significant interaction terms included sex: two involved sex × age interactions and one involved sex × group; no group × age interaction terms were significant. Given the number of comparisons, we only interpreted results with *p* < .001 or when the pattern of findings (*p*’s < .05) were consistent at ≥ 5 of 9 regions of interest within a frequency band.

Next, we computed partial correlations to investigate associations between power in each of the five frequency bands and the behavioral variables of verbal IQ, nonverbal IQ, social skills, and restricted/repetitive behaviors. Age was entered as a covariate for each, and we maintained a conventional *p*-value of .05 due to the exploratory nature of these analyses. Because our goal was to consider whether associations between resting EEG and clinical phenotype might differ by sex, we first considered the ASD group as a whole, and then for males and females separately.

## Results

### Sex

**Fig. 2:**
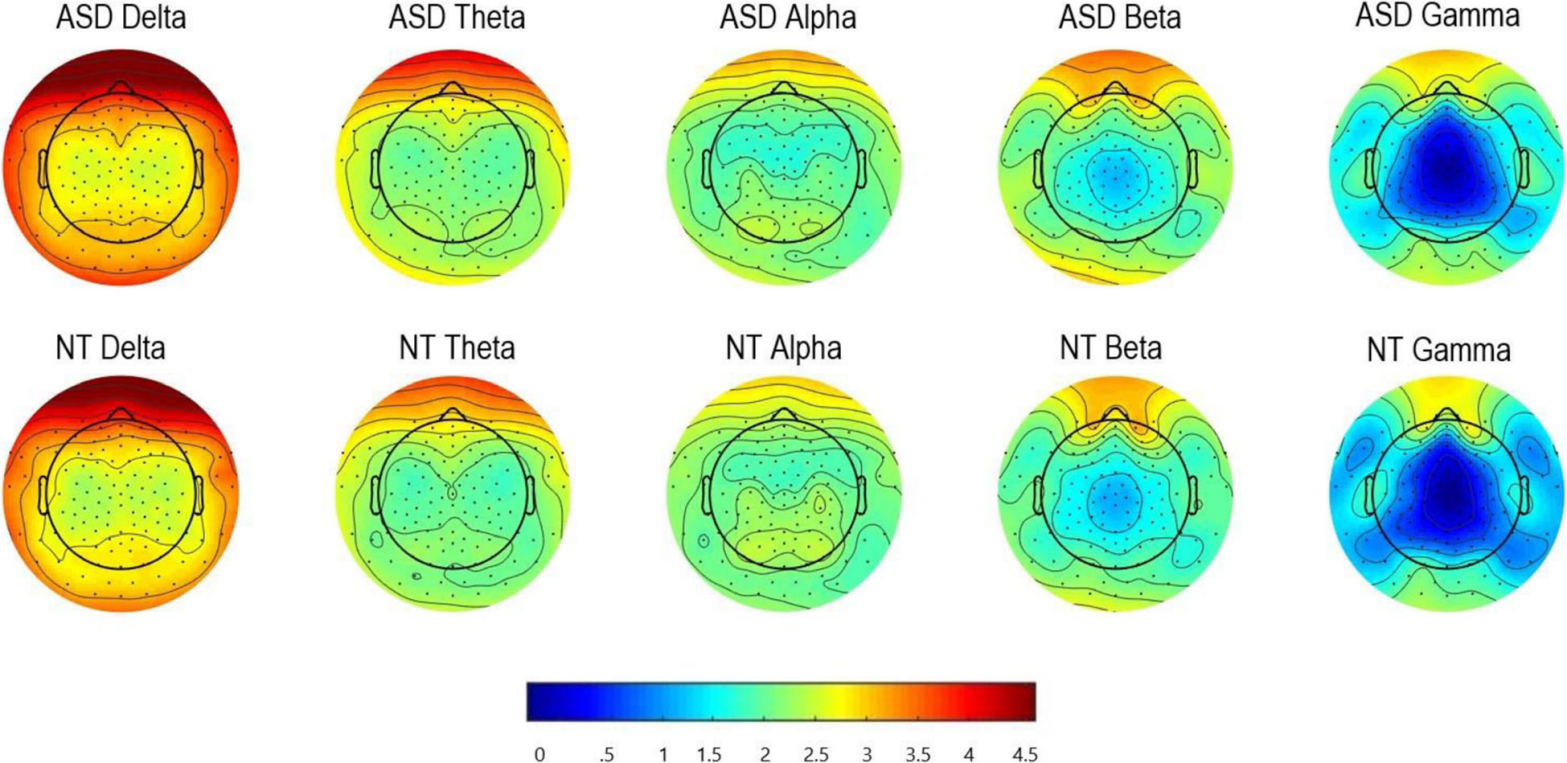
Topographical plots of absolute power (natural log-transformed) by frequency band and group. Colors depicted represent power (μV^2^) within frequency bands across regions. Blue areas correspond to lower power for a respective frequency band and red areas correspond to higher power

Model output is presented in Table 2, with topographical plots in Fig. 2. Differences by participant (biological) sex were apparent across all five frequency bands and across the entire scalp. Effects were more highly statistically significant in the central and posterior regions (*p*’s < .00001) with males compared to females having power values 10–57% greater in the central region and 34–61% greater in the posterior regions. Possible interactions between sex and age were observed in the anterior left region of interest in the two highest frequencies (beta: interaction term *p* = .045 and gamma: interaction term *p* = .020), suggesting that the relation between biological sex and power may vary by age in the anterior left region for higher frequencies. Specifically, for gamma, each additional year of age was associated with approximately 11% lower power in males, with no statistically significant difference in females. For beta, the same age effect was observed among males as for gamma; among females, each additional year of age was associated with a 6% lower beta power. These findings should be interpreted cautiously, given their *p*-values.

**Table 2:** Percent differences in EEG power associated with effects of ASD, sex, and age for the 5 frequency bands and by 9 regions of interest along the anterior to posterior gradient (anterior, central, and posterior) and hemisphere (left, midline [mid], and right).

		Delta			Theta			Alpha			Beta			Gamma		
		Left	Mid	Right	Left	Mid	Right	Left	Mid	Right	Left	Mid	Right	Left	Mid	Right
**ASD**																
*Anterior*	**% Diff**	− 4%	− 4%	− 4%	6%	6%	3%	**26%**	**20%**	**22%**	− 2%	− 2%	− 6%	− 5%	− 8%	− 12%
	***p*-val**	0.405	0.393	0.371	0.306	0.385	0.663	**0.001**	**0.011**	**0.004**	0.775	0.724	0.442	0.604	0.181	0.184
*Center*	**% Diff**	− 4%	0%	3%	8%	11%	14%	**36%**	**38%**	**46%**	2%	3%	5%	**(int.**^**2**^**)**	− 9%	− 15%
	***p*-val**	0.449	0.955	0.583	0.298	0.168	0.059	**0.001**	**0.001**	**< 0.001**	0.793	0.629	0.489		0.110	0.077
*Posterior*	**% Diff**	− 5%	2%	2%	3%	9%	12%	**23%**	**25%**	**35%**	0%	3%	7%	**− 16%**	− 10%	− 13%
	***p*-val**	0.416	0.738	0.790	0.676	0.248	0.141	**0.039**	**0.039**	**0.002**	0.946	0.669	0.371	**0.031**	0.230	0.094
**Male sex**																
*Anterior*	**% Diff**	**23%**	**15%**	**26%**	**17%**	7%	**19%**	15%	4%	**15%**	**(int.**^**1**^**)**	3%	**23%**	**(int.**^**3**^**)**	**− 14%**	10%
	***p*-val**	**0.001**	**0.004**	**< 0.001**	**< 0.001**	0.280	**0.001**	0.034	0.548	**0.025**		0.598	**0.007**		**0.018**	0.332
*Center*	**% Diff**	**44%**	**46%**	**43%**	**40%**	**50%**	**42%**	**39%**	**41%**	**57%**	**36%**	**35%**	**42%**	**(int.**^**2**^**)**	10%	**42%**
	***p*-val**	**< 0.001**	**< 0.001**	**< 0.001**	**< 0.001**	**< 0.001**	**< 0.001**	**< 0.001**	**< 0.001**	**< 0.001**	**< 0.001**	**< 0.001**	**< 0.001**		0.073	**< 0.001**
*Posterior*	**% Diff**	**46%**	**34%**	**42%**	**48%**	**36%**	**48%**	**58%**	**50%**	**61%**	**41%**	**39%**	**41%**	**44%**	**45%**	**41%**
	***p*-val**	**< 0.001**	**< 0.001**	**< 0.001**	**< 0.001**	**0.002**	**< 0.001**	**< 0.001**	**0.001**	**< 0.001**	**< 0.001**	**< 0.001**	**< 0.001**	**< 0.001**	**< 0.001**	**< 0.001**
**Age (years)**																
*Anterior*	**% Diff**	**− 11%**	**− 13%**	**− 12%**	**− 12%**	**− 13%**	**− 13%**	**− 8%**	**− 8%**	**− 10%**	**(int.**^**1**^**)**	**− 7%**	**− 10%**	**(int.**^**3**^**)**	**− 6%**	**− 7%**
	***p*-val**	**< 0.001**	**< 0.001**	**< 0.001**	**< 0.001**	**< 0.001**	**< 0.001**	**< 0.001**	**< 0.001**	**< 0.001**		**< 0.001**	**< 0.001**		**< 0.001**	**< 0.001**
*Center*	**% Diff**	**− 16%**	**− 12%**	**− 14%**	**− 16%**	**− 13%**	**− 16%**	**− 11%**	**− 8%**	**− 11%**	**− 8%**	**− 5%**	**− 8%**	**− 5%**	− 1%	**− 4%**
	***p*-val**	**< 0.001**	**< 0.001**	**< 0.001**	**< 0.001**	**< 0.001**	**< 0.001**	**< 0.001**	**< 0.001**	**< 0.001**	**< 0.001**	**< 0.001**	**< 0.001**	**0.003**	.107	**0.031**
*Posterior*	**% Diff**	**− 16%**	**− 14%**	**− 16%**	**− 16%**	**− 14%**	**− 16%**	**− 10%**	**− 10%**	**− 10%**	**− 8%**	**− 5%**	**− 8%**	**− 4%**	− 1%	**− 4%**
	***p*-val**	**< 0.001**	**< 0.001**	**< 0.001**	**< 0.001**	**< 0.001**	**< 0.001**	**< 0.001**	**< 0.001**	**< 0.001**	**< 0.001**	**< 0.001**	**< 0.001**	**0.002**	0.527	**0.014**

A separate linear mixed-effects model was run for each frequency-region combination. Models included ASD, age, and sex as covariates, and accounted for clustering by site and family. Participants ranged in age from 8 to 17 years

Interpretation for each estimate is as follows:

ASD: The percent difference in power associated with NO autism diagnosis, compared to children WITH an autism diagnosis, adjusting for ASD and sex

Sex: The percent difference in power associated with being male, compared to being female, adjusting for ASD and sex

Age: The percent difference in power associated with a 1-year increase in age, adjusting for ASD and sex

Int.1, int.2, int.3: interaction observed thus stratum specific estimates provided as follows:

1: For males, percent difference is − 10.8% for each additional year of age, vs −6.0% for females (both *p* < 0.001)

2: For males, percent difference is 4.2% for no ASD compared to ASD (not significant), vs − 28.0% for females (*p* = 0.015)

3: For males, percent difference is − 10.8% for each additional year of age (*p* < 0.001), vs − 2.4% for females (not significant)

Models which had significant interaction terms are thus indicated; otherwise interaction terms were not significant and therefore not included in the model

### Age

Findings indicated highly statistically significant differences by age, with power decreasing with each additional year of age. Specifically, power was estimated to be 3.6–15.6% lower additional year of age. As noted above, the two highest frequencies (anterior left region) showed interactions between sex and age (see sex-specific estimates above).

### Diagnostic group

As seen in Table 2, there were statistically significant differences by diagnosis for the alpha frequency only. For the alpha frequency, power at all nine regions of interest was 20–46% greater in NT youth compared to youth with ASD. For delta, theta, and beta, there were no group differences.

With respect to gamma power, we identified a possible interaction between diagnosis and sex at the left central region (*p* = .038), such that the relation between ASD diagnosis and power varied by sex. Specifically, among males, ASD was not associated with a statistically significant difference in power compared to NT, whereas among females, ASD was associated with 28% lower power compared to NT (*p* = .015). These latter findings should be interpreted particularly cautiously given the overall number of comparisons.

### Associations with phenotypic differences

Partial correlations for the ASD group (males and females combined) are presented in Table 3. As shown, a number of significant correlations emerged between parent-reported socialization as measured with the Vineland-II and power within the theta, alpha, and beta frequencies. Over and above the effects of age, participants with stronger socialization skills displayed less EEG power in all three bands. We did not observe significant correlations for verbal or nonverbal cognitive skills, nor for the restricted/repetitive symptom domain.

**Table 3:** Partial correlations between resting EEG and clinical phenotype for participants with ASD

		Delta			Theta			Alpha			Beta			Gamma		
		Left	Mid	Right	Left	Mid	Right	Left	Mid	Right	Left	Mid	Right	Left	Mid	Right
**Verbal IQ**																
*Anterior*	**Correlation**	.02	− .09	− .01	.10	.03	.03	.10	.06	.04	− .03	− .07	− .04	− .09	− .12	− .05
	***p*-val**	.83	.27	.90	.22	.74	.69	.24	.52	.62	.70	.38	.63	.28	.15	.58
*Center*	**Correlation**	.05	.02	− .03	.11	.04	.05	.10	.06	.06	.05	− .01	.02	.02	− .03	− .03
	***p*-val**	.53	.78	.71	.19	.63	.55	.25	.52	.49	.55	.92	.85	.79	.77	.69
*Posterior*	**Correlation**	.09	− .01	− .05	.15	.03	.00	.10	.05	.03	.08	.01	− .03	.02	.06	− .05
	***p*-val**	.30	.92	.58	.08	.68	.98	.22	.57	.73	.34	.94	.77	.78	.51	.54
**Nonverbal IQ**																
*Anterior*	**Correlation**	− .02	− .09	− .03	.00	− .03	− .04	− .11	− .13	− .12	− .13	− .16	− .13	− .14	− .16	− .10
	***p*-val**	.81	.28	.70	.98	.73	.65	.20	.13	.17	.12	.06	.12	.09	.06	.24
*Center*	**Correlation**	.00	− .02	− .11	.04	− .01	− .05	− .11	− .10	− .14	− .10	− .14	− .15	− .12	− .14	− .16
	***p*-val**	.96	.83	.20	.64	.88	.54	.21	.22	.10	.23	.09	.08	.15	.09	.06
*Posterior*	**Correlation**	.03	.03	− .06	.05	.01	− .06	− .07	− .04	− .14	− .09	− .06	− .15	− .15	.02	− .13
	***p*-val**	.70	.70	.50	.59	.89	.51	.41	.62	.09	.31	.50	.08	.08	.80	.12
**Vineland-II Socialization T-Score**																
*Anterior*	**Correlation**	− .11	− .14	− .01	**− .18**	− .17	− .10	**− .29**	**− .26**	**− .22**	**− .20**	**− .19**	− .10	− .09	− .12	− .05
	***p*-val**	.21	.12	.93	**.04**	.05	.28	**.00**	**.00**	**.02**	**.02**	**.04**	.26	.28	.15	.58
*Center*	**Correlation**	− .03	− .10	− .07	− .09	− .11	− .10	− .15	− .17	**− .18**	− .10	− .12	− .15	.02	− .03	− .03
	***p*-val**	.76	.29	.41	.31	.25	.27	.09	.06	.05	.26	.19	.09	.79	.77	.69
*Posterior*	**Correlation**	− .08	− .15	− .17	− .11	**− .24**	**− .22**	**− .22**	**− .28**	**− .29**	− .15	**− .25**	**− .26**	.02	.06	− .05
	***p*-val**	.40	.09	.06	.23	**.01**	**.01**	**.01**	**.00**	**.00**	.10	**.01**	**.00**	.78	.51	.54
**SRS-2 RBB T-Score**																
*Anterior*	**Correlation**	− .12	− .06	**− .19**	.02	− .04	− .09	.05	.03	− .01	.12	.07	.03	.17	**.18**	.09
	***p*-val**	.19	.51	**.04**	.84	.67	.34	.56	.79	.95	.19	.44	.71	.06	**.05**	.31
*Center*	**Correlation**	− .08	− .14	− .11	.00	− .11	− .09	.03	− .05	.00	.04	− .04	.00	.08	.03	.10
	***p*-val**	.36	.12	.24	1.00	.23	.35	.73	.59	.97	.66	.67	.97	.40	.74	.30
*Posterior*	**Correlation**	− .14	− .09	− .05	− .05	− .01	.01	.00	− .01	.01	.02	.04	.09	.09	.01	**.19**
	***p*-val**	.12	.35	.61	.63	.89	.91	1.00	.91	.89	.87	.66	.35	.35	.95	**.03**

Participant age was entered as a covariate in all correlations. *Mid* midline region

Female and male participants with ASD were then considered separately. Among females with ASD, phenotypic variables were largely unrelated to EEG power across frequencies (see Table 4 A). In contrast, for males with ASD, we again observed negative correlations between socialization as measured by the Vineland-II and power in the theta, alpha, and beta frequency bands. Possible associations were also noted between nonverbal IQ and gamma, as well as restricted/repetitive symptoms and gamma. These were such that higher gamma power during resting was associated with poorer nonverbal IQ and more restricted/repetitive symptoms (see Table 4 B). Thus, apparent associations between EEG power and phenotypic characteristics observed in the full ASD sample were driven by our male subgroup and did not reflect brain-behavior relations for our female youth with ASD.

**Table 4:** Subgroup partial correlations between resting EEG and clinical phenotype for (A) female youth with ASD and (B) male youth with ASD. Age was included as a covariate

		Delta			Theta			Alpha			Beta			Gamma		
		Left	Mid	Right	Left	Mid	Right	Left	Mid	Right	Left	Mid	Right	Left	Mid	Right
***A*.*Female ASD***																
**Verbal Standard Score**																
*Anterior*	**Correlation**	.10	.06	.14	.20	.12	.13	.08	.06	.00	.13	.02	.09	.09	− .02	.13
	***p*-val**	.43	.64	.27	.12	.36	.34	.54	.62	.99	.31	.88	.52	.50	.90	.34
*Center*	**Correlation**	.11	.13	.08	.18	.10	.20	.10	.04	.10	.09	.03	.07	.05	.01	− .02
	***p*-val**	.41	.31	.56	.17	.45	.13	.45	.79	.47	.48	.82	.61	.73	.93	.88
*Posterior*	**Correlation**	.16	.07	.05	.23	.09	.12	.13	.07	.05	.09	.04	.01	− .02	.02	− .07
	***p*-val**	.23	.60	.68	.07	.51	.35	.31	.60	.68	.52	.77	.92	.90	.87	.61
**Nonverbal Standard Score**																
*Anterior*	**Correlation**	− .06	− .06	.05	.04	.01	.01	− .10	− .11	− .12	.10	− .09	.08	.18	− .12	.17
	***p*-val**	.66	.67	.73	.79	.97	.91	.46	.38	.37	.47	.51	.52	.18	.37	.20
*Center*	**Correlation**	− .10	− .05	− .14	.01	− .03	− .06	− .12	− .15	− .15	− .07	− .19	− .15	.01	− .20	− .11
	***p*-val**	.44	.72	.28	.95	.82	.66	.36	.26	.25	.60	.15	.25	.94	.13	.39
*Posterior*	**Correlation**	.00	.02	− .12	.05	.04	− .05	− .06	− .01	− .13	− .10	− .13	− .18	− .15	− .12	− .18
	***p*-val**	.98	.89	.38	.72	.79	.68	.67	.97	.32	.44	.34	.16	.26	.37	.18
**Vineland-II Socialization T-Score**																
*Anterior*	**Correlation**	.01	− .05	.19	− .03	− .05	.08	− .18	− .15	− .11	− .25	− .17	− .03	**− .27**	− .24	− .07
	***p*-val**	.93	.70	.18	.81	.72	.56	.19	.28	.42	.07	.22	.83	**.04**	.08	.63
*Center*	**Correlation**	.03	.00	.06	.01	.02	.08	− .07	− .10	− .04	− .06	− .05	− .03	− .10	− .02	− .09
	***p*-val**	.84	.99	.65	.97	.88	.58	.62	.48	.78	.68	.72	.81	.48	.89	.50
*Posterior*	**Correlation**	.00	− .02	− .11	− .03	− .13	− .13	− .13	− .18	− .21	− .08	− .14	− .20	− .06	.09	− .19
	***p*-val**	.99	.87	.42	.84	.34	.34	.36	.20	.12	.57	.30	.14	.66	.51	.18
**SRS-2 RRB T-Score**																
*Anterior*	**Correlation**	− .14	− .14	− .26	− .08	− .12	− .17	− .03	− .02	− .06	.01	− .04	− .19	.13	.12	− .12
	***p*-val**	.32	.35	.07	.57	.39	.25	.85	.88	.67	.95	.77	.19	.37	.40	.39
*Center*	**Correlation**	− .16	− .14	− .13	− .11	− .11	− .15	− .09	.00	− .08	− .12	− .10	− .15	.05	.06	.01
	***p*-val**	.27	.33	.36	.46	.46	.30	.55	.99	.57	.42	.49	.30	.71	.67	.97
*Posterior*	**Correlation**	− .22	− .08	− .09	− .09	.02	− .03	− .05	.04	.01	− .07	.05	.01	.07	.08	.20
	***p*-val**	.13	.57	.52	.54	.87	.82	.74	.79	.94	.62	.76	.96	.61	.58	.17
***B*.*Male ASD***																
**Verbal IQ**																
*Anterior*	**Correlation**	− .01	− .17	− .08	.04	− .05	− .02	.12	.04	.07	− .12	− .13	− .12	**− .22**	− .21	− .17
	***p*-val**	.92	.13	.45	.70	.68	.87	.30	.71	.54	.29	.24	.28	**.05**	.06	.12
*Center*	**Correlation**	.09	− .03	− .10	.13	.01	− .03	.12	.08	.04	.04	− .02	− .03	− .02	− .06	− .09
	***p*-val**	.45	.82	.39	.27	.94	.77	.31	.49	.73	.71	.89	.80	.85	.59	.42
*Posterior*	**Correlation**	.08	− .04	− .10	.14	.02	− .06	.11	.04	.02	.10	.02	− .06	.03	.10	− .08
	***p*-val**	.46	.75	.39	.23	.84	.61	.34	.71	.84	.38	.86	.61	.79	.37	.51
**Nonverbal IQ**																
*Anterior*	**Correlation**	.00	− .11	− .10	− .04	− .07	− .09	− .13	− .15	− .13	**− .28**	− .21	**− .28**	**− .35**	− .19	**− .28**
	***p*-val**	.98	.33	.39	.71	.56	.43	.24	.19	.25	**.01**	.07	**.01**	**.00**	.09	**.01**
*Center*	**Correlation**	.09	.00	− .11	.06	− .02	− .07	− .10	− .09	− .16	− .14	− .13	− .19	**− .25**	− .11	**− .23**
	***p*-val**	.42	.97	.32	.57	.84	.52	.36	.45	.16	.22	.24	.10	**.03**	.32	**.04**
*Posterior*	**Correlation**	.06	.04	− .02	.04	− .02	− .08	− .10	− .09	− .18	− .09	− .02	− .15	− .18	.11	− .13
	***p*-val**	.63	.71	.85	.74	.88	.49	.39	.45	.11	.40	.89	.18	.11	.34	.25
**Vineland-II Socialization T-Score**																
*Anterior*	**Correlation**	− .24	− .23	− .16	**− .32**	**− .28**	− .23	**− .39**	**− .38**	**− .31**	− .17	− .21	− .13	− .08	− .10	− .06
	***p*-val**	.05	.06	.20	**.01**	**.02**	.06	**.00**	**.00**	**.01**	.16	.09	.28	.52	.43	.62
*Center*	**Correlation**	− .09	− .20	− .21	− .18	− .21	**− .25**	− .24	**− .24**	**− .31**	− .14	− .18	**− .25**	− .03	− .06	− .22
	***p*-val**	.48	.11	.09	.14	.09	**.04**	.05	**.05**	**.01**	.27	.14	**.04**	.79	.65	.07
*Posterior*	**Correlation**	− .15	**− .28**	− .22	− .19	**− .34**	**− .30**	**− .33**	**− .38**	**− .36**	− .22	**− .35**	**− .31**	− .15	− .21	**− .30**
	***p*-val**	.22	**.02**	.07	.13	**.00**	**.01**	**.01**	**.00**	**.00**	.07	**.00**	**.01**	.22	.09	**.01**
**SRS-2 RRB T-Score**																
*Anterior*	**Correlation**	− .03	.01	− .09	.17	.04	.02	.15	.07	.08	**.29**	.18	.24	**.27**	.20	**.26**
	***p*-val**	.81	.91	.46	.16	.72	.88	.21	.57	.54	**.02**	.15	.05	**.03**	.09	**.03**
*Center*	**Correlation**	.04	− .06	.02	.15	− .03	.06	.17	− .04	.15	.24	.10	.21	.16	.07	.24
	***p*-val**	.72	.62	.88	.23	.83	.63	.18	.77	.23	.05	.41	.08	.20	.59	.05
*Posterior*	**Correlation**	.00	− .02	.07	.07	.04	.14	.11	.01	.08	.21	.12	**.24**	.21	.03	**.29**
	***p*-val**	.99	.90	.54	.55	.75	.27	.36	.91	.49	.08	.33	**.05**	.09	.83	**.02**

## Discussion

Our findings yield a number of insights into resting state EEG among individuals with ASD, particularly with regard to its utility as a biomarker.

### Decreased alpha power in ASD

Consistent with expectations, we observed decreased alpha power in our ASD group compared to our NT group. Because alpha power is inversely related to neural activation, this suggests greater activation in the ASD group than in the NT group during a condition designed and assumed to capture a “resting” or baseline brain state. This finding could suggest that youth with ASD experience the experiment as an explicit task (e.g., complying with instructions to limit physical movements and attending to the screen) rather than as “rest” in an experimental sense. Indeed, Eilam-Stock et al. [49] suggest that when individuals with ASD participate in a resting activity in an experimental condition, the task of withdrawing attention from a preferred stimulus in order to comply with task demands could require high levels of effort. Thus, for some individuals, prompting and assessment of true “resting” activity that is unrelated to task or directions may be unlikely.

In addition to the effort elicited by task demands in general, the choice of resting condition and its specific characteristics likely affect EEG activity. Within EEG research, resting conditions vary across studies (e.g., eyes open with a fixation point, eyes open viewing videos, eye closed) [35, 37], with different implications for brain function. In the present study, we assessed resting state activity while participants viewed abstract dynamic images similar to screensavers. Our use of visual stimuli may have had a particular impact on observed alpha power, as recent work suggests that resting conditions with visual input elicit reduced alpha power relative to resting conditions without such input (e.g., eyes closed, eyes open in the dark) [50]. As such, reduced alpha power in our ASD group may indicate that the visual images presented during the resting condition elicited greater attention, interest, or cognitive engagement among our ASD group relative to our NT group.

Of note, an analogous discussion regarding resting state conditions exists within the fMRI literature, wherein direct comparisons of “eyes open” and “eyes closed” resting state conditions during fMRI demonstrate differences in functional connectivity among visual, salience, and default mode systems [51, 52]. Among ASD samples, “eyes open” resting states are associated with stronger local functional connectivity in posterior visual regions relative to “eyes closed” resting states, during which connectivity in subcortical and limbic regions appears stronger [53]. Moreover, greater test-retest reliability among “eyes open” resting states [54] and stronger associations between resting state activity and behavioral correlates [51] have led to suggestions that “eyes open” conditions may reduce variability across individuals and instances [53] and be better suited than “eyes closed” to the identification of behavioral correlates [51] — frequently an integral component of biomarker research.

### Role of development in decreasing power

Analyses also revealed developmental effects in the form of decreased power with increasing age across all frequency bands in our sample. Inspection of percentage of decrease with age (Table 2) indicates that the largest age-related decreases were among lower frequencies, with smaller deceases among higher frequencies. This pattern is consistent with literature documenting developmental shifts toward higher frequencies over time (e.g., [30–32]. Decreased absolute power has been linked to brain maturation (possibly from gray matter tissue loss), and hypothesized to relate to improved efficiency in information processing, cognitive capacity, and executive functioning [55, 56]. In our sample, in addition to robust main effects of age, we also observed interactions of age and sex for left frontal power in beta and gamma frequencies. Thus, sex differences in the developmental trajectories of high frequency EEG power are important to consider (e.g., [39]).

### Sex differences in EEG correlates

Our findings of decreased power across frequencies for females in our sample, as well as striking sex differences in the associations between EEG power and measures of cognitive, social, and behavioral functioning, also carry implications for the field’s understanding and implementation of biomarkers. Whereas males with ASD displayed lower theta and alpha power in the context of stronger social skills, these correlations were absent for females. Similarly, our data suggest possible links between gamma activity, nonverbal IQ, and restricted/repetitive difficulties among males, with no comparable links for females. These constructs — social, cognitive, and restricted/repetitive domains — are central to ASD, and the possibility that they relate differently to EEG measures across sexes suggests that biomarker studies that include only male samples, have insufficient statistical power to detect sex differences, or fail to analyze data for the possibility of sex differences in mixed samples, could define ASD biomarkers that misrepresent autistic females and thereby contribute to incorrect etiological models and ineffective clinical tools.

### Implications for treatment research

A central appeal of biomarkers relates to their use in intervention research, where they may serve as inclusion criteria for clinical trials identifying a more specific biotype of ASD, mechanistic targets for behavioral or pharmacological treatments, or indices that predict therapeutic change [2, 4, 57]. Our findings may support the potential utility of alpha power as a diagnostic or enrichment biomarker, as resting alpha power differed between our diagnostic groups, and increased neural activation during resting, at the individual level, may identify a more homogenous subgroup of autistic youth. Furthermore, alpha corresponded to social adaptive ability for *some* of our participants with ASD, and future research may test if alpha power predicts social functioning or social change. As alpha power also corresponds to neurocognitive features such as executive function and attention [18, 19], its utility in treatment research may extend beyond the variables measured here and could be relevant as an intermediate outcome for a wider array of important functional outcomes.

However, these implications come with a considerable caveat, as we found very little association between resting alpha and behavioral outcomes for females with ASD. Clinically-relevant biomarkers “must have *wide applicability* in a clinical population” [57], either through relevance to basic neural functioning or through association with clinical features. Biomarker-informed interventions that are developed, tested, or applied without clear evidence of validity in autistic girls and women may fail to achieve their intended function. The heterogeneity of ASD makes it particularly imperative that we intentionally and routinely seek out and evaluate the possibility of differential effects and associations across subsets of the ASD community, so as to understand the boundaries of biological models and to develop clinical tools that equitably serve the autistic community.

### Limitations and future research directions

Our current sample is comprised of individuals with relatively high cognitive skills, as inclusion required a score of 70 or higher on at least one domain of the DAS-II. Recent estimates indicate that approximately a third of children with ASD have cognitive skills below our inclusion threshold, falling within the range of intellectual disability [58]. As a result, we cannot quantify the extent to which our findings extend to individuals with both ASD and intellectual disability. Investigation of resting state EEG and its behavioral correlates among the full range of individuals with autism will be critical if biomarkers identified in some ASD samples are to generalize to the full spectrum. Fortunately, resting state tasks are well-suited for such work, as they impose fewer behavioral, verbal, and attentional demands than other neuroimaging methods [6].

Also prevalent among individuals with ASD are features of anxiety and ADHD (e.g., [59]), both of which appear to affect resting EEG power among populations without ASD [29]. Given their prevalence, efforts to define ASD biomarkers must consider the effect of comorbid disorders (and subthreshold features, in the absence of diagnosis) on physiology [2], as they likely represent critical sources of heterogeneity in the ASD population. Meta-analytic findings indicate that some resting state alterations may be transdiagnostic in nature [29], and other models suggest that alterations in lower frequencies might be common to numerous diagnostic groups, whereas alterations in higher frequencies might be specific to ASD [33]. Delineating points of convergence and divergence will necessitate models that consider ASD in the full context of associated diagnostic features (e.g., impulsivity, emotion dysregulation), particularly in relation to individuals’ sex, age, and other individual differences (e.g., [12]).

## Conclusion

In summary, findings presented here underscore the importance of approaching biomarker research from an inclusive perspective that (1) conceptualizes the neurobiology of ASD within its full heterogeneous nature and (2) incorporates moderators and individual differences into study designs with appropriate statistical and methodological rigor to identify scientifically and clinically meaningful differences within the ASD population. Our findings add to an emerging body of work suggesting differential biology-behavior associations by sex within ASD, often manifested as associations that hold only for males with ASD (e.g., [39]). An essential next step will be to move beyond such “null findings” for females with ASD, to identify and characterize associations for females with ASD, and to consider the implications of such sex differences in our etiological and clinical models of ASD broadly.

## Data Availability

The datasets generated and analyzed in the current study are available through the National Database for Autism Research (NDAR) study #2021.

## Footnotes

1 Of note, after data collection, EGI Inc. released an update notifying users of potential timing issues between Net Station 300 Amplifiers and the use of FireWire connected cameras. We dropped the first segment of each block of the experiment to account for potential timing offsets.

## References

[1] American Psychiatric Association (2013). Diagnostic and statistical manual of mental disorders.

[2] Jeste SS, Frohlich J, Loo SK (2015). Electrophysiological biomarkers of diagnosis and outcome in neurodevelopmental disorders. Curr Opin Neurol..

[3] Levin AR, Naples AJ, Scheffler AW, Webb SJ, Shic F, Sugar CA, Murias M, Bernier RA, Chawarska K, Dawson G, Faja S, Jeste S, Nelson CA, McPartland JC, Şentürk D, and the Autism Biomarkers Consortium for Clinical Trials (2020). Day-to-day test-retest reliability of EEG profiles in children with autism spectrum disorder and typical development. Front Integr Neurosci..

[4] McPartland JC, Bernier RA, Jeste SS, Dawson G, Nelson CA, Chawarska K (2020). The Autism Biomarkers Consortium for Clinical Trials (ABC-CT): scientific context, study design, and progress toward biomarker qualification. Front Integr Neurosci..

[5] Webb SJ, Shic F, Murias M, Sugar CA, Naples AJ, Barney E (2019). Biomarker acquisition and quality control for multi-site tudies: the Autism Biomarkers Consortium for Clinical Trials. Front Integr Neurosci..

[6] Webb SJ, Bernier R, Henderson HA, Johnson MH, Jones EJ, Lerner MD (2015). Guidelines and best practices for electrophysiological data collection, analysis and reporting in autism. J Autism Dev Disord..

[7] McVoy M, Lytle S, Fulchiero E, Aebi ME, Adeleye O, Sajatovic M (2019). A systematic review of quantitative EEG as a possible biomarker in child psychiatric disorders. Psychiatry Res..

[8] Wang J, Barstein J, Ethridge LE, Mosconi MW, Takarae Y, Sweeney JA (2013). Resting state EEG abnormalities in autism spectrum disorders. J Neurodev Disord..

[9] Kirschstein T, Köhling R (2009). What is the source of the EEG?. Clin EEG Neurosci..

[10] Pizzagalli D, JC, LCT, GGB (2007). Electroencephalography and high-density electrophysiological source localization. The Handbook of Psychophysiology. 3rd ed.

[11] Knyazev GG (2012). EEG delta oscillations as a correlate of basic homeostatic and motivational processes. Neurosci Biobehav Rev..

[12] Shephard E, Tye C, Ashwood KL, Azadi B, Asherson P, Bolton PF, McLoughlin G (2018). Resting-state neurophysiological activity patterns in young people with ASD, ADHD, and ASD + ADHD. J Autism Dev Disord..

[13] Penolazzi B, Spironelli C, Angrilli A (2008). Delta EEG activity as a marker of dysfunctional linguistic processing in developmental dyslexia. Psychophysiology..

[14] Twilhaar ES, Janssen TWP, de Kieviet JF, van Elburg RM, Oosterlaan J (2019). EEG profiles and associated neurodevelopmental outcomes after very preterm birth. Clin Neurophysiol..

[15] Van der Molen MJ, Van der Molen MW (2013). Reduced alpha and exaggerated theta power during the resting-state EEG in fragile X syndrome. Biol Psychol..

[16] Markovska-Simoska S, Pop-Jordanova N (2017). Quantitative EEG in children and adults with attention deficit hyperactivity disorder: comparison of absolute and relative power spectra and theta/beta ratio. Clin EEG Neurosci..

[17] Zimmermann R, Gschwandtner U, Wilhelm FH, Pflueger MO, Riecher-Rössler A, Fuhr P (2010). EEG spectral power and negative symptoms in at-risk individuals predict transition to psychosis. Schizophr Res..

[18] Ray WJ, Cole HW (1985). EEG alpha activity reflects attentional demands, and beta activity reflects emotional and cognitive processes. Science..

[19] Schürmann M, Başar E (2001). Functional aspects of alpha oscillations in the EEG. Int J Psychophysiol..

[20] Perry A, Stein L, Bentin S (2011). Motor and attentional mechanisms involved in social interaction--evidence from mu and alpha EEG suppression. Neuroimage..

[21] Neuper C, Pfurtscheller G (2001). Event-related dynamics of cortical rhythms: frequency-specific features and functional correlates. Int J Psychophysiol..

[22] Ahmed OJ, Cash SS (2013). Finding synchrony in the desynchronized EEG: the history and interpretation of gamma rhythms. Front Integr Neurosci..

[23] Chan AS, Sze SL, Cheung MC (2007). Quantitative electroencephalographic profiles for children with autistic spectrum disorder. Neuropsychology..

[24] Chan AS, Cheung MC, Han YM, Sze SL, Leung WW, Man HS (2009). Executive function deficits and neural discordance in children with autism spectrum disorders. Clin Neurophysiol..

[25] Cantor DS, Thatcher RW, Hrybyk M, Kaye H (1986). Computerized EEG analyses of autistic children. J Autism Dev Disord..

[26] Daoust AM, Limoges E, Bolduc C, Mottron L, Godbout R (2004). EEG spectral analysis of wakefulness and REM sleep in high functioning autistic spectrum disorders. Clin Neurophysiol..

[27] Stroganova TA, Nygren G, Tsetlin MM, Posikera IN, Gillberg C, Elam M, Orekhova EV (2007). Abnormal EEG lateralization in boys with autism. Clin Neurophysiol..

[28] Sutton SK, Burnette CP, Mundy PC, Meyer J, Vaughan A, Sanders C, Yale M (2005). Resting cortical brain activity and social behavior in higher functioning children with autism. J Child Psychol Psychiatry..

[29] Newson JJ, Thiagarajan TC (2018). EEG frequency bands in psychiatric disorders: a review of resting state studies. Front Hum Neurosci..

[30] Clarke AR, Barry RJ, McCarthy R, Selikowitz M (2001). Age and sex effects in the EEG: development of the normal child. Clin Neurophysiol..

[31] Matousek M, Petersén I (1973). Automatic evaluation of EEG background activity by means of age-dependent EEG quotients. Electroencephalogr Clin Neurophysiol..

[32] Anderson AJ, Perone S (2018). Developmental change in the resting state electroencephalogram: Insights into cognition and the brain. Brain Cogn..

[33] Cornew L, Roberts TP, Blaskey L, Edgar JC (2012). Resting-state oscillatory activity in autism spectrum disorders. J Autism Dev Disord..

[34] Vakorin VA, Doesburg SM, Leung RC, Vogan VM, Anagnostou E, Taylor MJ (2017). Developmental changes in neuromagnetic rhythms and network synchrony in autism. Ann Neurol..

[35] Maxwell CR, Villalobos ME, Schultz RT, Herpertz-Dahlmann B, Konrad K, Kohls G (2015). Atypical laterality of resting gamma oscillations in autism spectrum disorders. J Autism Dev Disord..

[36] Orekhova EV, Stroganova TA, Nygren G, Tsetlin MM, Posikera IN, Gillberg C, Elam M (2007). Excess of high frequency electroencephalogram oscillations in boys with autism. Biol Psychiatry..

[37] Mathewson KJ, Jetha MK, Drmic IE, Bryson SE, Goldberg JO, Schmidt LA (2012). Regional EEG alpha power, coherence, and behavioral symptomatology in autism spectrum disorder. Clin Neurophysiol..

[38] Happé F, Frith U (2006). The weak coherence account: detail-focused cognitive style in autism spectrum disorders. J Autism Dev Disord..

[39] Brito NH, Elliott AJ, Isler JR, Rodriguez C, Friedrich C, Shuffrey LC, Fifer WP (2019). Neonatal EEG linked to individual differences in socioemotional outcomes and autism risk in toddlers. Dev Psychobiol..

[40] Rutter ML, Le Couteur A, Lord C (2003). Autism diagnostic interview - revised.

[41] Lord C, Rutter M, DiLavore PC, Risi S, Gotham K, Bishop S (2012). Autism Diagnostic Observation Schedule, second edition (ADOS-2) manual (part I): modules 1–4.

[42] American Psychiatric Association (2000). Diagnostic and statistical manual of mental disorders: DSM-IV-TR.

[43] Constantino JN (2012). Social Responsiveness Scale, second edition (SRS-2).

[44] Rutter ML, Bailey A, Lord C (2003). Social Communication Questionnaire.

[45] Elliott CD (2007). Differential Ability Scales, 2nd edition.

[46] Sparrow S, Cicchetti D, Balla D (2005). Vineland Adaptive Behavior Scales, 2nd ed.

[47] Frohlich J, Senturk D, Saravanapandian V, Golshani P, Reiter LT, Sankar R (2016). A quantitative electrophysiological biomarker of duplication 15q11.2-q13.1 syndrome. PLoS One.

[48] McEvoy K, Hasenstab K, Senturk D, Sanders A, Jeste SS (2015). Physiologic artifacts in resting state oscillations in young children: methodological considerations for noisy data. Brain Imaging Behav..

[49] Eilam-Stock T, Xu P, Cao M, Gu X, Van Dam NT, Anagnostou E (2014). Abnormal autonomic and associated brain activities during rest in autism spectrum disorder. Brain..

[50] Webster K, Ro T. Visual modulation of resting state alpha oscillations. eNeuro. 2020;7(1):1–12. 10.1523/ENEURO.0268-19.2019. 10.1523/ENEURO.0268-19.2019 PMC6944479 31836596

[51] Agcaoglu O, Wilson TW, Wang YP, Stephen J, Calhoun VD (2019). Resting state connectivity differences in eyes open versus eyes closed conditions. Hum Brain Mapp..

[52] Weng Y, Liu X, Hu H, Huang H, Zheng S, Chen Q, Song J, Cao B, Wang J, Wang S, Huang R (2020). Open eyes and closed eyes elicit different temporal properties of brain functional networks. Neuroimage..

[53] Nair S, Jao Keehn RJ, Berkebile MM, Maximo JO, Witkowska N, Muller RA (2018). Local resting state functional connectivity in autism: site and cohort variability and the effect of eye status. Brain Imaging Behav..

[54] Patriat R, Molloy EK, Meier TB, Kirk GR, Nair VA, Meyerand ME, Prabhakaran V, Birn RM (2013). The effect of resting condition on resting-state fMRI reliability and consistency: a comparison between resting with eyes open, closed, and fixated. Neuroimage..

[55] Kharitonova M, Martin RE, Gabrieli JD, Sheridan MA (2013). Cortical gray-matter thinning is associated with age-related improvements on executive function tasks. Dev Cogn Neurosci..

[56] Tamnes CK, Walhovd KB, Grydeland H, Holland D, Østby Y, Dale AM, Fjell AM (2013). Longitudinal working memory development is related to structural maturation of frontal and parietal cortices. J Cogn Neurosci..

[57] McPartland JC (2017). Developing clinically practicable biomarkers for autism spectrum disorder. J Autism Dev Disord..

[58] Baio J, Wiggins L, Christensen DL, Maenner MJ, Daniels J, Warren Z, Kurzius-Spencer M, Zahorodny W, Robinson C, Rosenberg, White T, Durkin MS, Imm P, Nikolaou L, Yeargin-Allsopp M, Lee LC, Harrington R, Lopez M, Fitzgerald RT, Hewitt A, Pettygrove S, Constantino JN, Vehorn A, Shenouda J, Hall-Lande J, van K, Naarden, Braun, Dowling NF (2018). Prevalence of autism spectrum disorder among children aged 8 years - Autism and Developmental Disabilities Monitoring Network, 11 Sites, United States, 2014. MMWR Surveill Summ..

[59] Neuhaus E, Webb SJ, Bernier RA (2019). Linking social motivation with social skill: the role of emotion dysregulation in autism spectrum disorder. Dev Psychopathol..

